# Ibuprofen inhibits anaplastic thyroid cells in vivo and in vitro by triggering NLRP3-ASC-GSDMD-dependent pyroptosis

**DOI:** 10.1007/s10787-023-01379-7

**Published:** 2023-11-24

**Authors:** Haohao Guo, Runsheng Ma, Yifei Zhang, Keyu Yin, Gongbo Du, Fanxiang Yin, Hongqiang Li, Ziyang Wang, Detao Yin

**Affiliations:** 1 Department of Thyroid Surgery, The First Affiliated Hospital of Zhengzhou University, Zhengzhou, 450052 Henan China; 2 Engineering Research Center of Multidisciplinary Diagnosis and Treatment of Thyroid Cancer of Henan Province, Zhengzhou, 450052 Henan China; 3 Key Medicine Laboratory of Thyroid Cancer of Henan Province, Zhengzhou, 450052 Henan China; 4 Translational Medical Center, The First Affiliated Hospital of Zhengzhou University, Zhengzhou, 450052 Henan China; 5 School of Basic Medical Sciences, Lanzhou University, Lanzhou, 730000 Gansu China

**Keywords:** Ibuprofen, Anaplastic thyroid cancer, Pyroptosis, Inflammation

## Abstract

Pyroptosis is a novel type of proinflammatory programmed cell death that is associated with inflammation, immunity, and cancer. Anaplastic thyroid carcinoma (ATC) has a high fatality rate, and there is no effective or standard treatment. The disease progresses rapidly and these tumors can invade the trachea and esophagus, leading to breathing and swallowing difficulties. Hence, new treatment methods are greatly needed. Ibuprofen is a common drug that can exert antitumor effects in some cancers. In this study, we demonstrated in vitro and in vivo that ibuprofen can induce ATC pyroptosis. Hence, we treated C643 and OCUT-2C ATC cells with ibuprofen and found that several dying cells presented the characteristic morphological features of pyroptosis, such as bubble-like swelling and membrane rupture, accompanied by activation of ASC and NLRP3 and cleavage of GSDMD. Along with the increased release of LDH, ibuprofen treatment promoted apoptosis and inhibited viability, invasion, and migration. However, overexpression of GSDMD significantly inhibited ibuprofen-induced pyroptosis. In vivo, research has demonstrated that thyroid tumor growth in nude mice can be suppressed by ibuprofen-induced pyroptosis in a dose-dependent manner. In this research, we explored a new mechanism by which ibuprofen inhibits ATC growth and progression and highlighted its promise as a therapeutic agent for ATC.

## Introduction

Thyroid cancer is a common cancer that accounts for 90% of endocrine-related neoplasms (Li et al. 2019). Among thyroid cancers, PTC (papillary thyroid carcinoma) has a high incidence but seldom induces death, but ATC has the opposite characteristics: it has a low incidence and a high fatality rate (Megwalu and Moon 2022; Saini et al. 2018). At the cellular level, ATC is characterized by multinucleated cells with large singular nuclei, multiple classical features of mitosis, and no signs of thyroid differentiation (O'Neill and Shaha 2013). Thus, the median survival time for ATC is 3–5 months, and the 1-year survival rate is 10–20% (Are and Shaha 2006; Kebebew et al. 2005; Nagaiah et al. 2011). According to the American Thyroid Association (ATA), surgical resection is used for the first-line treatment of ATC, and external beam radiation therapy is used for local control. Although total thyroidectomy with high-dose radiation therapy improves survival, approximately 15–20% of ATC patients will experience recurrence in their lifetime. Currently, there is no effective treatment for ATC (Glaser et al. 2016; Sasanakietkul et al. 2018). We are currently searching for a new method to treat or control it.

Ibuprofen (a nonsteroidal anti-inflammatory drug) has anti-inflammatory, analgesic, and antipyretic properties due to its inhibition of cyclooxygenase-1 and-2 (COX-1/2) activities (Jin et al. 2022; Sakr et al. 2021). Epidemiological, clinical, and laboratory studies have shown that ibuprofen can inhibit the proliferation and induce the apoptosis of cancer cells (Khwaja et al. 2004; Pennock et al. 2018; Schack et al. 2019; Shen et al. 2020). NSAIDs may also promote the antitumor effect of chemotherapy and radiotherapy (Akrami et al. 2018). Previous studies have shown that NSAIDs work against cancer by inhibiting COX-2, which is highly expressed in cancer (Shen et al. 2020). However, an increasing body of evidence suggests that NSAIDs lacking COX inhibitory activity in cells still have significant anticancer effects. An interesting phenomenon is that ibuprofen can increase NLRP3 expression to induce pyroptosis in this research, which was the opposite of our common hypothesis. Therefore, ibuprofen has a new mechanism that requires further exploration. Next, we will elaborate on how ibuprofen induces pyroptosis and explore a new anticancer mechanism.

Pyroptosis is a nontraditional programmed death pathway that is similar to apoptosis and represents inflammatory lytic cell death pathways (Jiang et al. 2020; Tsuchiya 2021). Pyroptosis includes the classical inflammasome pathway and the nonclassical inflammasome pathway. The classic inflammasome pathway involves the recognition of DAMPs or PAMPs via pattern recognition receptors (PRRs), which activate the cytoplasmic signaling complex of the inflammasome, leading to pyroptosis (Alu et al. 2020). Classical pyroptosis is associated with the NLRP3 inflammasome complex, which contains proteins such as NLRP3, apoptosis-associated speck-like protein (ASC), and caspase-1 (Mouasni et al. 2019). Procaspase-1 activated by the inflammasome cleaves GSDMD to generate GSDMD-N fragments that can induce pyroptosis by forming 1–2 nm pores in the cell plasma membrane (Dubois et al. 2019). However, the role of pyroptosis in the antitumor effects of ibuprofen in ATC, and its underlying mechanism remain unclear.

In this study, we found that ibuprofen induces pyroptosis in anaplastic thyroid cancer cells via the NLRP3/ASC/GSDMD pathway, which activates the NLRP3 inflammasome and cleaves GSDMD in vitro and in vivo. However, overexpressing GSDMD abolished the activating effect of ibuprofen on NLRP3/ASC/GSDMD pathway-mediated pyroptosis and reversed the suppressive effect of ibuprofen on biological function in vitro. The in vivo results suggested that ibuprofen inhibits thyroid tumor growth by inducing pyroptosis, and ibuprofen at 20 mg/kg had a better antitumor effect than the control group. In conclusion, ibuprofen induces pyroptosis by triggering the NLRP3/ASC/GSDMD pathway, and these findings may facilitate the development of new strategies for ATC treatment.

## Materials and methods

### Cell culture

The ATC cell line C643 was purchased from Procell Life Science & Technology Co., Ltd., and OCUT-2C cells were purchased from iCell Bioscience Inc., Shanghai. C643 cells were cultured in RPMI 1640 (Gibco), and OCUT-2C cells were cultured in DMEM (Gibco), medium with 10% fetal bovine serum (FBS), and 1% penicillinstreptomycin solution (100 μg/ml, Leagene Biotech Co., Ltd.). The cells were incubated at 37 °C in a humidified 5% CO_2_ atmosphere. Ibuprofen (purity ≥ 99.93%) was purchased from MedChemExpress and was directly dissolved in RPMI 1640 or DMEM. Ibuprofen was dissolved the day before, and the solution was put in a water bath at 37 ℃ to aid in the dissolving process.

### CCK8 assays

C643 and OCUT-2C cells were seeded in 96-well plates (5 × 103 cells/well) in a complete medium and cultured overnight. Next, different concentrations of ibuprofen were administered to the different groups for 48 h. Then, 10 µl of Cell Counting Kit-8 (CCK-8) assay reagent (Dojindo, Kumamoto, Japan) was added to each well, and the 96-well plates were cultured at 5% CO_2_ at 37 °C for 2 h. The absorbance was measured at 450 nm using a microplate reader. The experiment was repeated three times for each well. The viability rate of control cells was set as 100% for the analysis.

### Transwell

The 24-well culture plate, including transwell upper chamber inserts, was used for transwell invasion assays (Corning, New York, USA). First, 1 × 104 cells or cells transfected with OE-RNA and/or control vectors were mixed with 100 ml serum-free RPMI-1640 or DMEM with different concentrations of ibuprofen (C643 at 0, 0.5, 1, and 1.5 mM, and OCUT-2C at 0, 1, 2, and 3 mM) added to the upper chamber. Then, 600 ml of medium containing 10% FBS with the same concentrations of ibuprofen was added to the lower chamber. After culture at 5% CO_2_ at 37 °C for 48 h, the chamber was removed from the plates and fixed with 4% paraformaldehyde for 30 min. The cells that traversed through the membrane pores were stained with crystal violet. Ultimately, the number of cells passing from the upper chamber to the lower chamber was observed through an inverted microscope (Olympus, Tokyo, Japan).

### Wound healing assay

The cells were seeded in a 6-well plate at 4 × 105 cells or transfected with OE-RNA and/or control vectors cultured overnight. Then, the cell surface was scratched using a 200 µl sterile pipette when the cells reached > 95% confluence. Then, the suspended cell fragments were removed by washing with PBS buffer, and the cells were cultured in serum-free RPMI-1640 or DMEM for 48 h with different concentrations of ibuprofen. Finally, the ability of cells to migrate was assessed using an inverted microscope (Olympus, Tokyo, Japan).

### Flow cytometry

Apoptosis was analyzed via flow cytometry. The cells or cells transfected with OE-RNA and/or control vectors were spread in a 6 cm dish at a density of 4 × 105 cells/well and cultured overnight. Next, the cells were treated with different concentrations of ibuprofen for 48 h. Then, the cells floating in the medium were collected, washed three times with PBS buffer, and digested with EDTA-free trypsin. Then, the obtained cells were labeled with PI and Annexin V (Jiangsu Keygen Biotech Corp, Ltd). Finally, a flow cytometer was used for analysis. Double-positive (PI and Annexin V) cells were considered apoptotic.

### LDH

Cells were seeded in a 6 cm dish at a density of 30 × 104 cells/well. Then, the cells were cultured with ibuprofen at different concentrations for 48 h. The supernatant of cells or cells transfected with OE-RNA and/or control vectors with different concentrations of ibuprofen was added to a 96-well plate. Distilled water, pyruvate standard solution, matrix buffer, and coenzyme I were added to the sample test well and the control well, and the cells were cultured at 37 °C for 15 min. After, 2,4-dinitrophenylhydrazine was added to each well and cultured at 37 °C for 15 min. Then, 0.4 mol/l NaOH solution was added, and the cells were cultured at 37 °C for 5 min. The absorbance at 450 nm was measured using a microplate reader. Then, the LDH content difference was calculated according to the obtained results.

### Transmission electron microscopy (TEM)

Cells were seeded in a 10 cm dish at a density of 80 × 104 cells/well. Then, cells were treated with different concentrations of ibuprofen (C643 at 0.1.5 mM and OCUT-2C at 0.3 mM) for 48 h. Next, the cells were fixed with 2.5% glutaraldehyde according to the manufacturer’s protocol (Beijing Solarbio Science & Technology Co., Ltd.). Then, the samples were dehydrated with acetone and embedded in epoxy resin and hardener. Finally, the samples were cut into ultrathin sections via a Recheron ultrathin microtome, stained with uranium acetate, and observed by electron microscopy.

### Immunofluorescence

Cells were seeded in a 96-well plate at a density of 5 × 103 cells/well. First, the cells were treated with different concentrations of ibuprofen (C643 at 0.1.5 mM and OCUT at 0.3 mM) for 48 h. Next, the cells were fixed with 4% paraformaldehyde and then permeabilized with 0.5% Triton X-100. Then, the cells were incubated with the primary antibody at 4 °C overnight, and then the secondary antibody was added and incubated for 1 h. Immunofluorescence was performed with the following antibodies: rabbit anti‐ASC (Servicebio, GB115270), rabbit anti‐NLRP3 (SAB, 29125), rabbit anti‐GSDMD (Proteintech, 20770-1-AP), goat Anti-Rabbit IgG (abbkine, A23220) for Dylight 488, and Goat Anti-Rabbit IgG (abbkine,A23620) for Dylight 649. DAPI (Solarbio, S2110) was used as a nuclear counterstain. The images were observed with a Nikon Ti Eclipse Confocal Microscope and an LSM 980 using basic operating techniques.

### OE-RNA transfection

The lentiviral OE-GSDMD vectors and their control vectors were constructed by Shanghai GenePharma Co., Ltd. All transfections were performed according to the manufacturer’s instructions. C643 and OCUT-2C cells were plated in six-well plates (1 × 10^5^ cells/well). Then, 5 µl of OE-GSDMD or control vectors was transfected with Lipofectamine 3000 (Invitrogen) based on the manufacturer’s protocol. After 24 h, transfected C643 and OCUT-2C cells were treated with ibuprofen and subjected to subsequent analyses.

### Western blotting

RIPA buffer was used to lyse cells for protein extraction at 4 °C. Equal amounts of protein (20 μl) were added to 10% SDS/PAGE and transferred to a PVDF membrane. Then, the cells were blocked with a quick block solution for 15 min at room temperature and washed three times with TBST buffer. The membrane was incubated with the primary antibodies at 4 °C overnight, and then the secondary antibody was added and incubated for 1 h. Western blotting was performed with the following antibodies: rabbit anti‐ASC (Servicebio, GB115270), rabbit anti‐NLRP3 (SAB, 29125), rabbit anti‐GSDMD (Proteintech, 20770-1-AP), rabbit anti‐GAPDH (Good here, AB-P-R001), and goat anti‐rabbit IgG (Dingguo, IH-0011). The blots were visualized with an enhanced chemiluminescence kit (Servicebio) based on the manufacturer’s instructions.

### Xenograft models

A 200 µl cell suspension containing 2 × 106 cells was subcutaneously injected into 5-week-old female BALB/c nude mice (Beijing Vital River Laboratory Animal Technology Co., Ltd.). After the tumors grew to approximately 0.5 mm^3^, 15 mice were randomly divided into the following 3 groups: the control group, in which mice were given 0.9% saline intragastrically, and the ibuprofen groups, in which mice were given 10 or 20 mg/kg ibuprofen. All groups were given solutions once every two days. The tumor volumes were monitored using a digital caliper. The xenograft tumor volume (mm^3^) was calculated as 0.5 × (shortest diameter)^2^ × (longest diameter). When some tumors had grown to about 2 mm^3^, the mice were killed and the size and weight of the xenograft tumors were measured.

### Immunohistochemistry analysis and hematoxylin–Eosin (HE) staining

The xenografts tumor, normal thyroid tissues, and stomach were separated from mice and fixed in 4% paraformaldehyde buffer for HE staining or immunohistochemistry staining. The operation procedure of this animal experiment was carried out under the program approved by the Ethics Committee on Laboratory Animal Management of Zhengzhou University.

### Statistical analysis

GraphPad Prime 9.0 was used for mapping and statistical analysis of the experimental data. Measurement data are expressed as the mean ± standard deviation (mean ± SD). Paired *T*-test and one-way ANOVA were used for statistical analysis. *P* < 0.05 indicated a statistical difference between the samples.

## Results

### Ibuprofen can inhibit thyroid cancer cell growth and induce pyroptosis

**Fig. 1:**
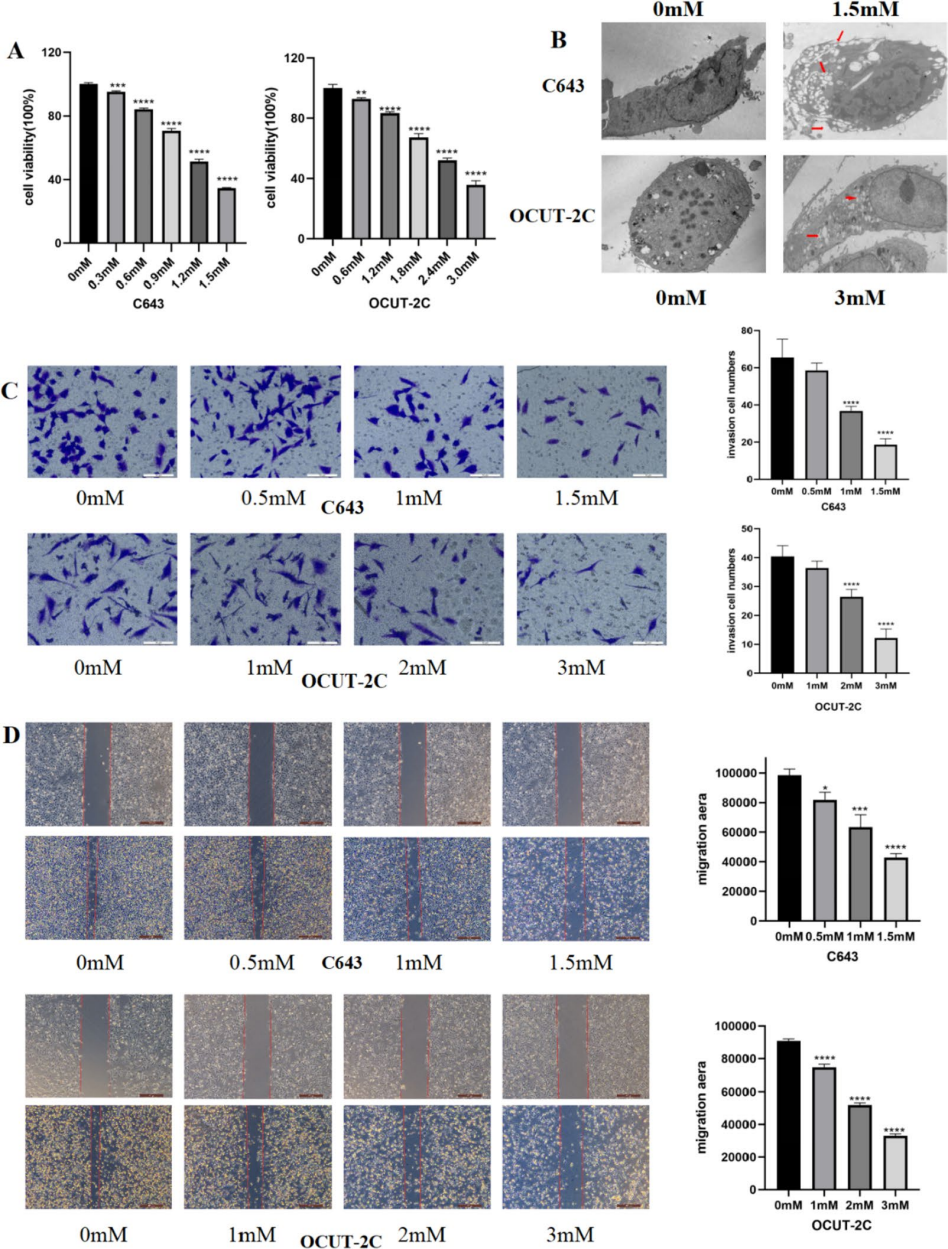
Ibuprofen induces pyroptosis in anaplastic thyroid cancer cells. **A** The inhibitory effect of ibuprofen on the proliferation of C643 and OCUT-2C cells was detected by CCK8 assay. **B** The features of pyroptosis in C643 and OCUT-2C cells were detected by TEM. **C** Transwell assay, cells were treated with various concentrations of ibuprofen (C643 at 0, 0.5, 1, and 1.5 mM; OCUT-2C at 0, 1, 2, and 3 mM) for 48 h view cell invasion abilities. **D.** Wound healing assay, cells were treated with different concentrations of ibuprofen (C643 at 0, 0.5, 1, and 1.5 mM; OCUT-2C at 0, 1, 2, and 3 mM) for 48 h view cell migration abilities. The data are expressed as the means ± SDs, * *P* < 0.05, ** *P* < 0.01, *** *P* < 0.001

To determine the viability of anaplastic thyroid cancer cells treated with ibuprofen, C643 and OCUT-2C cells were treated with different concentrations of ibuprofen for 48 h, and CCK8 assays were performed. The results showed that ATC cell proliferation was inhibited by increasing ibuprofen concentrations in the treatment groups compared with the control group (Fig. 1A). Next, we selected three effective drug concentrations (C643 at 0.5, 1, and 1.5 mM and OCUT-2C at 1, 2, and 3 mM) and a control group for the following relevant experiments. To further clarify the type of cell death induced by ibuprofen, transmission electron microscopy (TEM) was used to observe the cell morphology. The morphological changes of the cells in the ibuprofen group were very obvious. Ibuprofen can induce cell pore formation on the cell membrane, cell swelling, and a low density of cytosol, but the nucleus remains intact, which is a significant morphological sign of pyroptosis. Bubble-like swelling and membrane rupture are indicated by red arrows (Fig. 1B). The results suggested that ibuprofen can inhibit cell growth and induce cell pyroptosis.

### Ibuprofen inhibits thyroid cancer cell invasion and migration, promotes cell LDH release, and induces the apoptosis pathway

**Fig. 2:**
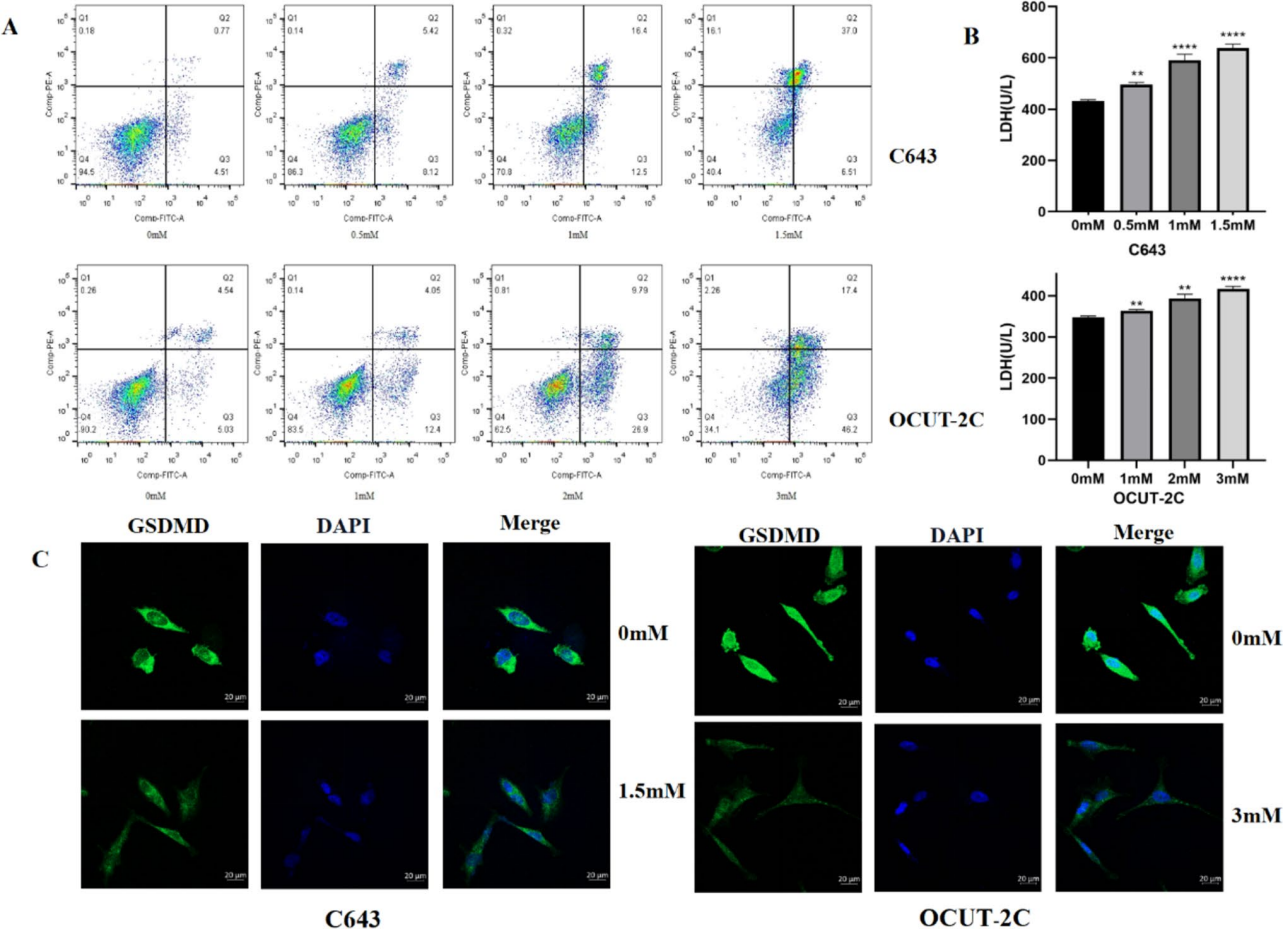
Ibuprofen induces pyroptosis in anaplastic thyroid cancer cells. **A** Flow cytometry analysis of ATC cells treated with different ibuprofen concentrations for 48 h stained with Annexin V-FITC and PI. **B** The effect of ibuprofen on LDH activity in C643 and OCUT-2C cells. **C** Immunofluorescence detection of the expression of GSDMD in C643 and OCUT-2C cells. The data are expressed as the means ± SDs,* *P* < 0.05, ** *P* < 0.01, *** *P* < 0.001

To research whether ibuprofen could inhibit the abilities of invasion and migration of anaplastic thyroid cancer cells, we performed transwell assays and wound healing assays to test whether ibuprofen influences the invasion and migration of ATC cells in vitro. The results showed that the invasive (Fig. 1C) and migratory (Fig. 1D) abilities of cells were markedly decreased with increasing ibuprofen concentrations. Subsequently, the breakdown of the plasma membrane resulted in the release of LDH into the culture supernatants. We measured the LDH in the cell supernatant and found that LDH was increased gradually in a concentration-dependent manner (Fig. 2B). Then, to investigate whether ibuprofen induced cell apoptosis, we performed flow cytometry to assess the proportion of cells double-positive for Annexin V-FITC and PI. The results showed that with increasing ibuprofen concentrations, the apoptosis rate of ATC cells gradually increased (Fig. 2A).

### Ibuprofen-induced thyroid cancer cell pyroptosis is mediated by the ASC/NLRP3/GSDMD pathway

**Fig. 3:**
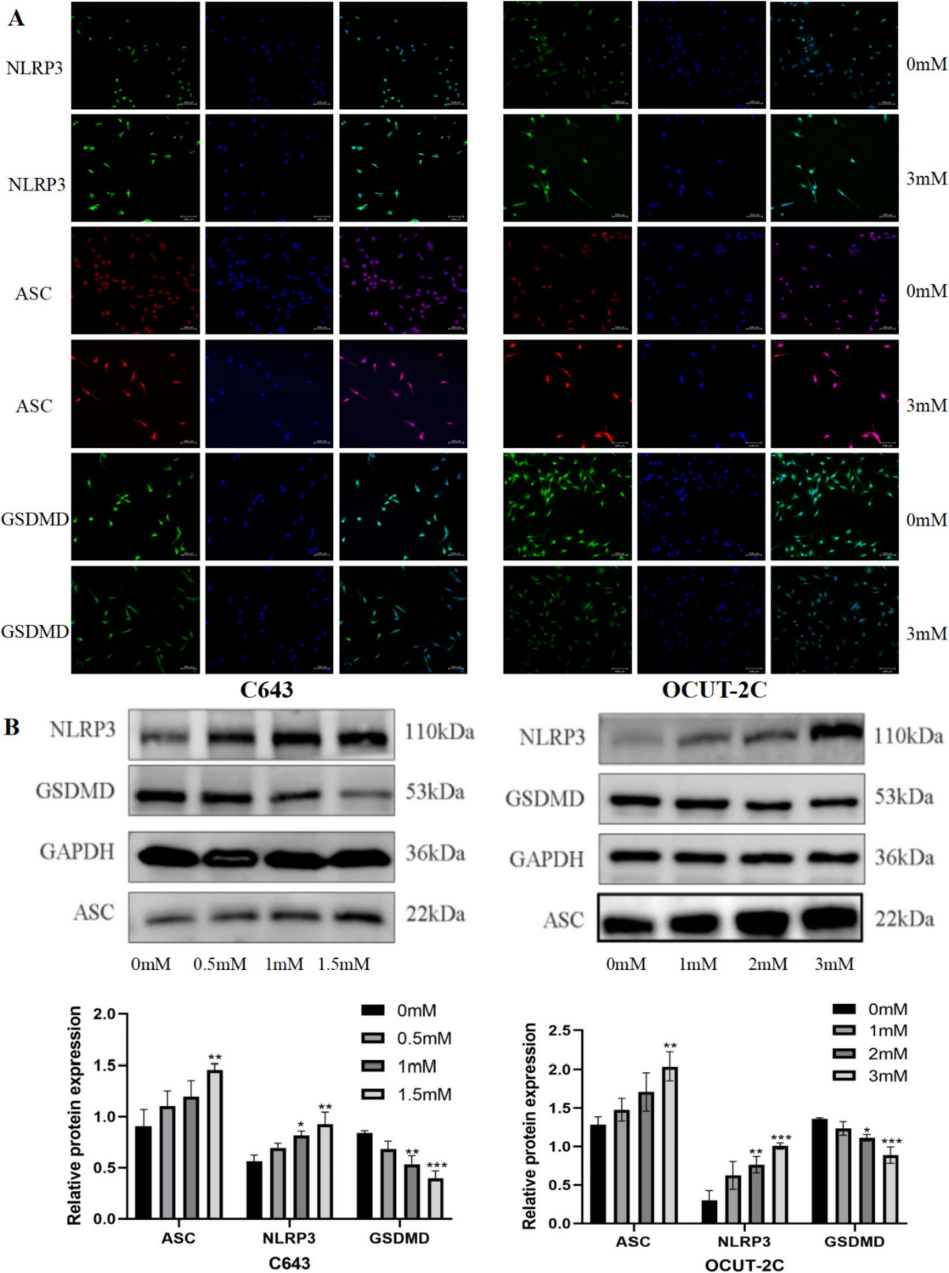
Ibuprofen leads to pyroptosis in anaplastic thyroid cancer cells is induced by ASC/NLRP3/GSDMD pathway. **A.** Immunofluorescence detection of ASC, NLRP3, and GSDMD expression in C643 and OCUT-2c cells. **B** Representative western blot analysis of ASC, NLRP3, and GSDMD in C643 and OCUT-2c cells. The data are expressed as the means ± SDs, * *P* < 0.05, ** *P* < 0.01, *** *P* < 0.001

We used different concentrations of ibuprofen to treat ATC cells to explore the mechanism of cell pyroptosis. The NLRP3 inflammasome is an intracellular protein complex that is an important mediator of inflammation in many pathologies. Activation of the NLRP3 inflammasome usually leads to proinflammatory programmed cell death, and this process is known as pyroptosis (Coll et al. 2022). As a key regulator of inflammation and cell death, the role of ASCs in innate immunity and immunoinflammatory diseases is becoming increasingly clear. ASC is the adaptor protein of the inflammasome, including the N-terminal PYD and C-terminal CARD. Activation of NLRP3 can induce ASC formation. GSDMD plays a major role in pyroptosis, and cleaved GSDMD induces pyroptosis and leads to the release of proinflammatory cellular contents (Burdette et al. 2021). Therefore, we explored whether NLRP3, ASC, and GSDMD were involved in the pyroptosis of ATC cells induced by ibuprofen. Western blot analysis showed that NLRP3 and ASC were increased and GSDMD was decreased (Fig. 3B). The immunofluorescence assay was implemented to further verify protein expression. The results showed that ibuprofen increased NLRP3 and ASC expression, and GSDMD deficiency as well as western blot. In particular, GSDMD was uniformly distributed in the cytoplasm before ibuprofen treatment and membrane foci appeared after drug treatment (Figs. 2C, 3A). The data suggested that ibuprofen induces pyroptosis by activating the NLRP3-ASC-GSDMD pathway in ATC cells.

### GSDMD overexpressed expression can significantly suppress ibuprofen-induced cell pyroptosis

**Fig. 4:**
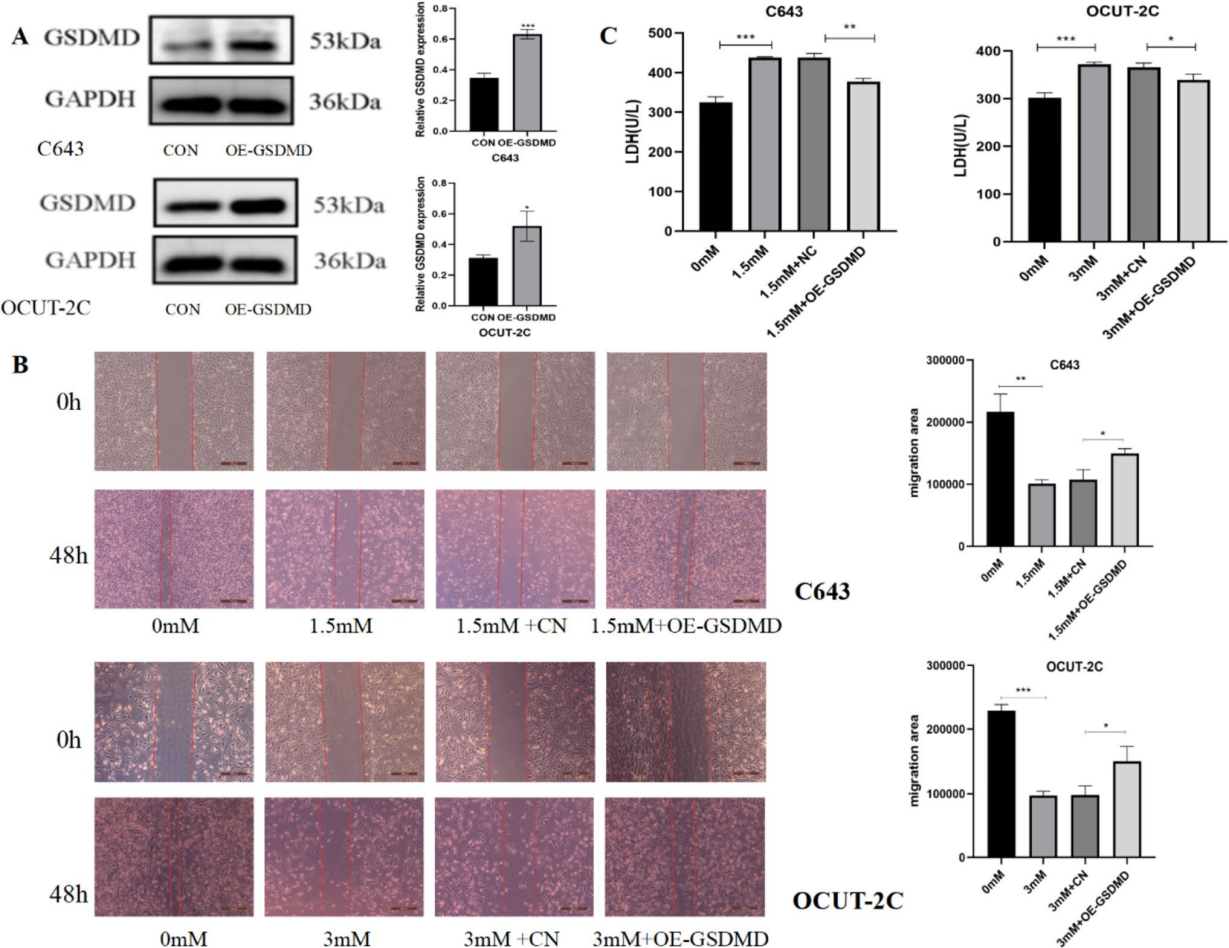
GSDMD overexpression inhibited ibuprofen-induced pyroptosis. **A** The protein expression of GSDMD in C643 and OCUT-2C cells. **B** Wound healing, cells were treated with the four groups of C643 and OCUT-2C cells for 48 h to observe cell migration abilities. **C.** Effect of ibuprofen on LDH activity in C643 and OCUT-2C cells (0 mM group, ibuprofen group, ibuprofen + CN group, ibuprofen + OE-GSDMD group). The data are expressed as the means ± SDs, * P < 0.05, ** *P* < 0.01, *** *P* < 0.001

**Fig. 5:**
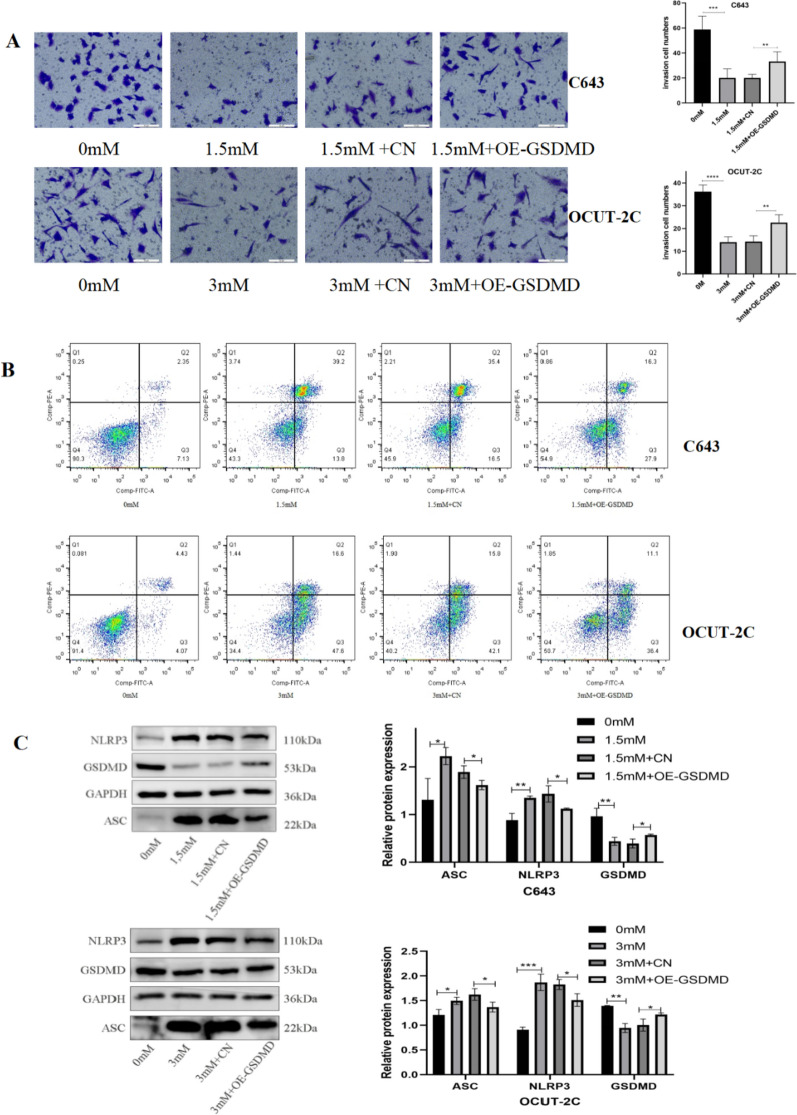
Overexpression of GSDMD significantly enhanced thyroid cancer cell resistance to ibuprofen. **A** Transwell assay, cells were treated with the four groups of C643 and OCUT-2C cells for 48 h to observe cell migration. **B** Flow cytometry analysis of ATC cells treated with different ibuprofen groups for 48 h stained with Annexin V-FITC and PI. **C** Representative western blot analysis of ASC, NLRP3, and GSDMD. The data are expressed as the means ± SDs, * *P* < 0.05, ** *P* < 0.01, *** *P* < 0.001

According to the above results, ibuprofen can induce ATC cell pyroptosis via activation of the NLRP3/ASC/GSDMD pathway. Thus, we overexpressed GSDMD in ATC cells to evaluate the role of GSDMD in pyroptosis. Next, we examined whether GSDMD overexpression influences cell biological function and protein expression. The Wb results showed that GSDMD was highly expressed in cells transfected with plasmids (Fig. 4A). Transwell assays and wound healing assays showed that overexpression of GSDMD effectively alleviated the inhibitory effect of ibuprofen on cell invasion (Fig. 5A) and migration (Fig. 4B). In addition, we found that the increase in LDH release induced by ibuprofen was inhibited in the OE-GSDMD + ibuprofen treatment group (Fig. 4C), suggesting that GSDMD overexpression may decrease the damage to the cell membrane integrity caused by ibuprofen. Moreover, flow cytometry analysis showed that the increase in the percentage of double-positive annexin V and PI cells was eliminated in the OE-GSDMD + ibuprofen treatment group (Fig. 5B), suggesting that the activation of pyroptosis by ibuprofen was blocked by GSDMD overexpression. Next, we examined whether expression changed at the protein level by western blotting, and the results showed that NLRP3 and ASC levels decreased, while GSDMD levels increased in the overexpression + ibuprofen group compared with the ibuprofen group (Fig. 5C). Considering all these results, we speculated that GSDMD overexpression significantly enhances ATC cell resistance to ibuprofen.

### Ibuprofen inhibits the growth of xenograft tumors by inducing pyroptosis of thyroid cells

**Fig. 6:**
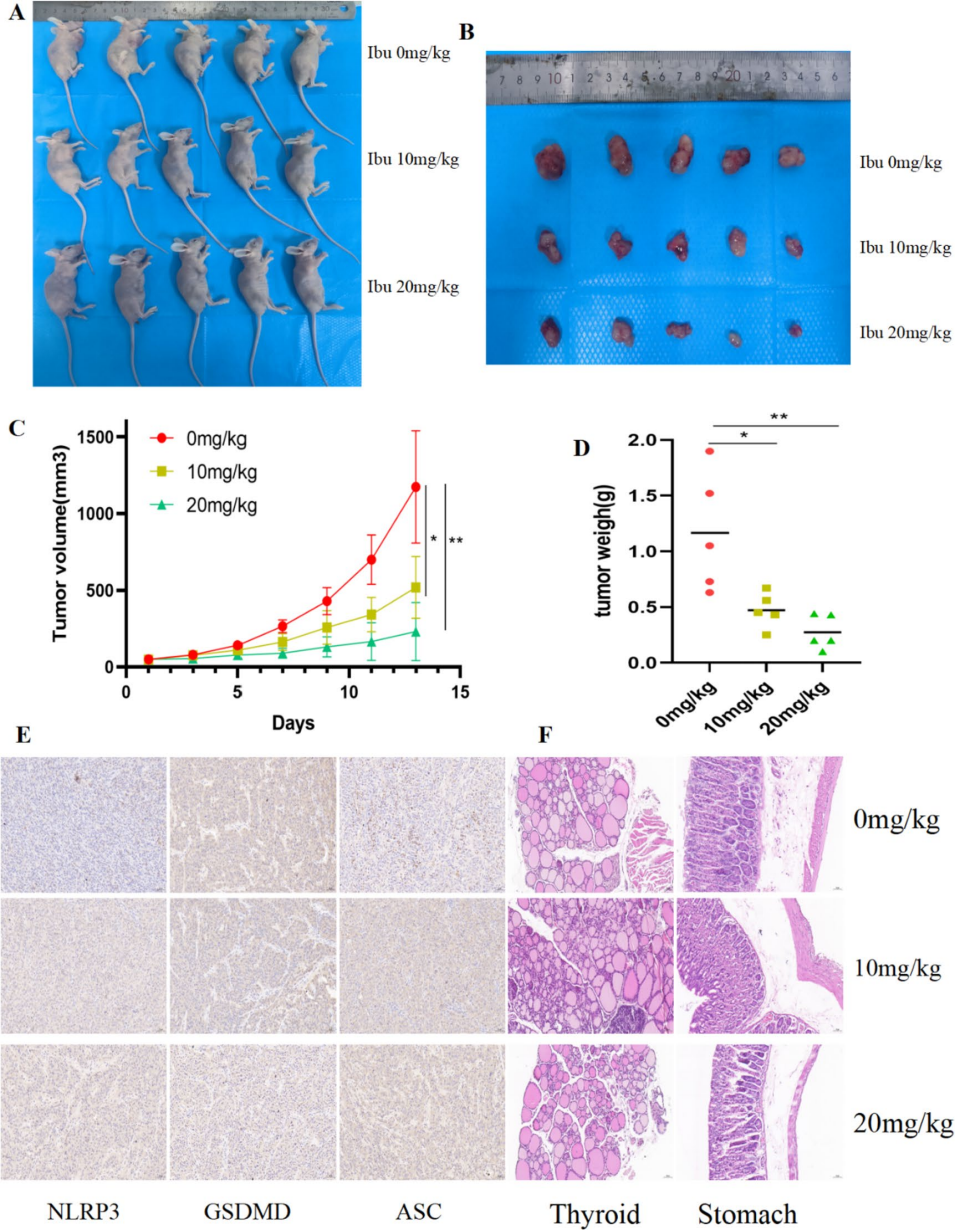
Ibuprofen potently inhibits the tumorigenicity of OCUT-2C cells in vivo. **A** Mouse body weight, **B** Tumor volume, **C** Tumor growth curve, **D** Tumor weight, **E** Ibuprofen influences the pyroptosis protein levels of ASC, NLRP3, and GSDMD in mice. The expression levels of ASC, NLRP3, and GSDMD proteins in xenograft tumors were measured by IHC staining. **F** HE staining for normal thyroid and stomach

In vitro, experiments showed that ibuprofen can induce ATC cell pyroptosis through the NLRP3-ASC-GSDMD pathway. To further confirm the effect of ibuprofen on the formation and growth of anaplastic thyroid tumors in vivo, we injected OCUT-2C ATC cells (2 × 10^6^ cells/mouse) subcutaneously into BALB/c nude female mice. After tumor formation in mice, ibuprofen was administered at different concentrations (0, 10, and 20 mg/kg) to test its ability to inhibit tumor growth. The ATC cell-derived tumors were dissected after the part of ATC cells to grow at 2 mm^3^. Compared with the control group, the ibuprofen treatment group markedly inhibited tumor growth and reduced volume (Fig. 6A–D). However, there was no damage to normal thyroid tissue and stomach (Fig. 6F). Then, we researched the effect of ibuprofen on pyroptosis-related proteins and inflammatory factors in tumor tissues. The IHC suggested that the expression of NLRP3 and ASC increased, and the expression of GSDMD decreased in the ibuprofen group (Fig. 6E). In conclusion, ibuprofen can inhibit the growth of anaplastic thyroid xenograft tumors by inducing cancer cell pyroptosis.

## Discussion

Anaplastic thyroid cancer is a solid tumor with high invasiveness and a high mortality rate; thus, it seriously threatens the lives of people worldwide (Erickson 2021; Jin and Kim 2021). The treatment of thyroid cancer includes surgical therapy, chemoradiotherapy, immunotherapy, and targeted therapy. However, due to the strong invasion of the tumor and its insensitivity to radiotherapy and chemotherapy, the clinical treatment effect of anaplastic thyroid cancer is poor. Hence, it is necessary for us to explore effective treatments to control or treat ATC. In the previous research, we found that ibuprofen can induce cancers to generate ferroptosis or apoptosis in cancers to inhibit tumor proliferation and invasion abilities. In this study, we used two ATC cell lines, C643 and OCUT-2C, to research the effect and molecular mechanism of ibuprofen in the treatment of ATC. Our research indicated that ibuprofen induced pyroptosis in ATC.

The pyroptosis of cancer cells indicates that it plays a key role in tumorigenesis (Xia et al. 2019). Pyroptosis is a pro-inflammatory type of programmed cell death that involves the gasdermin family, consisting of six members, namely, gasdermin A–E and DFNB59. It is characterized by the formation of pores in the cell membrane, which disrupts the balance of the ion gradients on both sides of the cell membrane, resulting in water influx, cell swelling, and the release of cellular pro-inflammatory cytokines. Therefore, pyroptosis has been recognized as a type of inflammatory “necrosis”. GSDMD is expressed in immune cells and esophageal, gastric, and intestinal epithelial cells. GSDMD is an executor of the gasdermin family that can be cleaved to GSDMD-N and GSDMD-C, and cells continue to expand until the cell membrane bursts, resulting in the release of cell contents that activate intense inflammatory and immune responses (Wang et al. 2020). NLRP3 and ASC belong to the NLRP3 inflammasome complex. ASC, as a connector protein, contains the PYD and CARD as domains, while NLRP3, as a receptor protein, contains three domains including the PYD. The PYD domain of ASC can interact with the PYD domain of NLRP3. Inflammasome formation leads to pyroptosis (Guo et al. 2023).

Ibuprofen is a common anti-inflammatory drug in clinical practice, with fever-reducing, anti-inflammatory, analgesic and other effect. It inhibits cyclooxygenase to inhibit the production of prostaglandin (PG) to exert its effects (Moore et al. 2014). In addition, previous studies have demonstrated that NSAIDs may be able to prevent the development and proliferation of tumors. Research has shown that ibuprofen can be used to treat gastric and prostate cancer by inducing apoptosis and inhibiting the proliferation and metastasis of cancer cells, but the anti-cancer effects are independent of the cyclooxygenase mechanism (Akrami et al. 2018; Stabile et al. 2018; Upadhyay et al. 2021). Ibuprofen inhibits glioma cell viability by inducing ferroptosis in glioma cells and reduces cell proliferation by inhibiting the Wnt signaling pathway in gastric cancer stem cells (Akrami et al. 2018; Gao et al. 2020). The anti-cancer ability of ibuprofen has aroused widespread interest in exploring the clinical benefits of ibuprofen in cancer treatment (Dandah et al. 2018). Therefore, its anti-cancer mechanism needs to be further explored.

In the current study, we demonstrated that ibuprofen inhibited ATC mainly by inducing pyroptosis. Ibuprofen can decrease the invasion and migration abilities of ATC cells. Migration and invasion experiments clearly showed that the toxicity of drugs to cells also affects their migration and invasion ability. Ibuprofen can lead to morphological changes in ATC cell membrane rupture, bubble-like characteristics and cell shrinking of the nuclears. Cell membrane rupture results in the release of LDH into the extracellular space. Ibuprofen activated NLRP3, ASC, and cleaving GSDMD to induce cell pyroptosis, but overexpression of GSDMD effectively attenuated the ibuprofen-induced damage to ATC cells. GSDMD overexpression rescued the phenotype, but the damage levels were not significantly different from that in the control group. This suggests that there may be other pathways involved. For example, the Wnt/β pathway that inhibits gastric cancer stem cells or induces ferroptosis in glioma may be involved in the inhibition of ATC by ibuprofen, but this study aimed to explore the pyroptosis pathways. Additionally, the ATC cell OCUT-2C xenograft mice model was used to examine the effects of ibuprofen on tumor cell suppression in vivo. Compared with the control group, the ibuprofen group showed significant inhibition of tumor growth, but ibuprofen had no effect on normal thyroid tissue. However, ibuprofen has side effects, including the potential development of gastric ulcers. In our study, we found that ibuprofen has anticancer activity without causing gastric injury. In vitro and in vivo studies have shown that ibuprofen inhibits ATC cell proliferation by inducing pyroptosis. These data provide direct evidence that ibuprofen activates the NLRP3-ASC-GSDMD axis to induce pyroptosis in ATC cells, emphasizing a prospective therapeutic strategy.

From a clinical point of view, among all NSAIDs, ibuprofen is the least damaging one to the gastric mucosa, and patients who take large doses of ibuprofen over the years have fewer side effects (Konstan et al. 1995; Warner et al. 1999). Therefore, long-term use of ibuprofen as an analgesic may underlie provide effective cancer prevention in patients. Since ibuprofen can be spread throughout the body through the blood, it may have a potential use in chemoprevention and cancer treatment (Albert et al. 1984; Khwaja et al. 2004).

To summarize, ibuprofen may be a potential therapeutic agent for ATC that acts by inducing pyroptosis of tumors by cleaving GSDMD. The results of this study provide an experimental and theoretical basis for the treatment of anaplastic thyroid cancer cells with ibuprofen and for the clinical treatment of ATC. Hence, we need to further explore the mechanism of action of ibuprofen in ATC and investigate multiple oncogenic mechanisms by exploring key proteins in the signaling pathway to facilitate its clinical application in ATC.

## Data Availability

Not applicable.

## Abbreviations

ATC: Anaplastic thyroid cell
ASC: Apoptosis speck-like protein (ASC)
NLRP3: NOD-like receptor thermal protein domain associated protein 3
PRR: Contain pattern-recognition receptors
PAMPs: Pathogen-associated molecular patterns
DAMPs: Damage-associated molecular patterns
GSDMD-NT: The N-terminal domain of GSDMD
